# Effects of Different Vegetation-Soil Conditions on Soil Physicochemical Properties, Enzyme Activities, and Microbial Communities in Bauxite Mine Wasteland of Southwest China

**DOI:** 10.3390/plants15172560

**Published:** 2026-08-23

**Authors:** Guangxu Zhu, Xingyun Zhao, Yunyan Wang, Yakun Zhang, Yunhe Zhao, Qiang Tu

**Affiliations:** 1 College of Biology and Environment Engineering, Guiyang University, Guiyang 550005, China; 18865395798@163.com (X.Z.); shihuang0308@163.com (Y.W.); 15081601981@163.com (Y.Z.); 2 Center for Environmental Remediation, Institute of Geographic Sciences and Natural Resources Research, Chinese Academy of Sciences, Beijing 100101, China; zhaoyunhe@stu.ynu.edu.cn; 3 Helmholtz International Lab for Anti-Infectives, Shandong University–Helmholtz Institute of Biotechnology, State Key Laboratory of Microbial Technology, Shandong University, Qingdao 266237, China; 4 Institute of Synthetic Biology Industry, Hunan University of Arts and Science, Changde 415000, China

**Keywords:** bauxite mine wasteland, vegetation restoration, soil improvement, microbial community, Southwest China, karst region

## Abstract

Bauxite mining causes severe soil degradation in the ecologically fragile karst region of Southwest China, yet targeted vegetation restoration schemes remain poorly developed. This study aimed to screen promising selected restoration species and preliminarily explore their soil improvement characteristics in karst bauxite mine wastelands through a field observational survey. We investigated six vegetation-soil conditions at 0–10 cm topsoil (two-year monocultures of *Robinia pseudoacacia*, *Ligustrum lucidum*, *Zea mays*, *Miscanthus sinensis*, topsoil from >15-year *Z. mays* with straw return, and unvegetated bare land), alongside paired 10–20 cm subsoil from the same long-term *Z. mays* stand as a depth-profile reference, and analyzed soil physicochemical properties, enzyme activities, and bacterial/fungal communities via high-throughput sequencing. Results showed that vegetation cover neutralized strongly acidic mine soil and significantly increased soil organic matter (SOM), total nitrogen (TN), and available nutrients compared with the bare control (*p* < 0.05). The long-term *Z. mays* cropland showed the strongest soil nutrient accumulation, with topsoil SOM, available phosphorus, and available potassium, increased by 428.6%, 250.0%, and 65.3%, respectively, relative to the bare control. Notably, the subsoil of long-term *Z. mays* also maintained high nutrient levels, but its absolute values are not directly comparable with topsoil treatments due to different sampling depths. All vegetation conditions elevated soil enzyme activities and microbial alpha diversity and significantly shifted bacterial and fungal community structure (beta diversity) compared with the control. Several bacterial phyla (including Chloroflexi and Proteobacteria) that have been associated with carbon-cycling functions in prior studies were relatively more abundant in revegetated soils; however, their functional roles in this system remain to be verified. Redundancy analysis identified SOM, TN, and available potassium as key environmental correlates of bacterial community variation. Overall, the long-term crop–straw return pattern shows comprehensive soil improvement effects, providing observational baseline data and reference for ecological restoration of karst bauxite mining areas in Southwest China.

## 1. Introduction

Bauxite is a critical strategic mineral resource and the primary feedstock for the global aluminum industry, underpinning industrial development and economic security [1]. As one of China’s core bauxite-producing provinces, Guizhou ranks third nationwide in proven reserves, with high-quality diaspore deposits concentrated in contiguous mining areas central to China’s aluminum industrial chain [2]. Located in the core zone of the Southwest China karst region, Guizhou has an inherently fragile ecological environment characterized by shallow soil layers, low environmental carrying capacity, and poor self-recovery capacity after disturbance. Long-term open-pit bauxite mining has caused irreversible ecological damage, including complete vegetation removal, soil structural collapse, severe nutrient depletion, and amplified risks of soil erosion and slope instability, posing persistent threats to regional ecological security [3,4,5]. Accordingly, developing targeted ecological restoration technologies for karst bauxite mine wastelands has become an urgent priority for regional environmental governance.

Current mine restoration technologies include engineering, physical, chemical, and vegetation-based approaches. Engineering and physicochemical remediation can achieve short-term soil improvement, but their large-scale application in mountainous wastelands is constrained by high costs and potential secondary contamination [6]. In contrast, vegetation restoration is widely recognized as the most cost-effective and sustainable strategy for mine land reclamation, as it delivers long-term ecosystem services including soil conservation, carbon sequestration, and biodiversity support [7]. Guided by ecological succession theory, vegetation restoration rebuilds soil–plant systems by selecting stress-tolerant species, thereby progressively improving soil fertility and enhancing ecosystem stability [8]. Accumulating evidence shows that vegetation restoration not only replenishes soil nutrient pools but also modulates microbial community assembly by supplying root exudates and litter, which in turn accelerates biogeochemical cycling [9,10,11].

While integrated studies combining soil physicochemical properties, enzyme activities, and high-throughput sequencing have been widely conducted in various restoration ecosystems, three critical knowledge gaps remain for karst bauxite wastelands. First, most studies focus on coal and metal mines in northern China, whereas research on bauxite wastelands in the karst mountains of Southwest China remains scarce, with no region-specific restoration schemes. Second, many evaluations are confined to soil physicochemical properties, and the coupled responses of soil properties, enzyme activities, and microbial communities under different vegetation patterns remain poorly understood. Third, restoration species selection often lacks systematic comparison of selected species with different functional traits; few studies have evaluated the soil improvement effects of species across different life forms (nitrogen-fixing trees, fast-growing crops, pioneer grasses) under identical site conditions in karst bauxite areas [12,13]. Unlike previous studies focusing on single species or single indicators, this study integrates physicochemical, enzymatic, and high-throughput sequencing approaches to fill this knowledge gap.

Four selected species with contrasting functional traits are promising candidates for mine restoration in the Guizhou karst region: the nitrogen-fixing tree *Robinia pseudoacacia*, the stress-tolerant evergreen tree *Ligustrum lucidum*, the high-biomass crop *Zea mays*, and the pioneer herb *Miscanthus sinensis* [14,15,16,17]. However, their comparative performance and soil improvement characteristics in bauxite mine wastelands remain largely untested, especially the vertical distribution of soil properties under long-term continuous *Z. mays* cultivation with straw return. This study selected a typical bauxite mine wasteland in Qingzhen, Guizhou, as the study site and systematically analyzed soil physicochemical properties, enzyme activities, and microbial community characteristics across different vegetation-soil conditions. The specific objectives were: (1) to quantify and compare the soil properties under different selected vegetation conditions in the mining wasteland; (2) to reveal the correlations among soil properties, enzyme activities, and microbial communities and identify key environmental correlates; and (3) to discuss preliminary restoration implications for karst bauxite mine wastelands based on observational findings.

## 2. Results

### 2.1. Soil Physicochemical Properties Under Different Vegetation-Soil Conditions

The basic soil physicochemical properties across different vegetation-soil conditions are summarized in Table 1. The unvegetated control (CK) in the bauxite mine wasteland was strongly acidic (pH = 5.67) with extremely low organic matter and nutrient contents, reflecting severe soil quality degradation at the site. By contrast, all vegetation cover conditions significantly improved most soil physicochemical properties, with significant differences in improvement efficacy among patterns (*p* < 0.05).

Soil pH in all topsoil vegetation conditions was significantly higher than that in CK (*p* < 0.05), shifting from strongly acidic to neutral or weakly alkaline. The YM condition had the highest pH (8.07), followed by CH (7.89) and BC (7.74), while BT (6.77) and NZ (6.92) had relatively lower values.

Soil organic matter (SOM) content in topsoil followed the order BT > NZ > CH = BC > YM > CK. The BT condition had the highest topsoil SOM content (17.55 g/kg); it was 428.6% higher than CK and significantly greater than all other two-year conditions (*p* < 0.05). NZ (9.22 g/kg) also had significantly higher SOM than CH, BC, and YM. The ST subsoil of long-term maize contained 38.87 g/kg SOM, indicating substantial vertical nutrient accumulation under long-term straw return management; however, this value is not directly comparable with topsoil treatments due to different sampling depths.

For total nutrients, YM had the highest total nitrogen (TN) content (12.60 g/kg), a 524.6% increase relative to CK (2.02 g/kg), followed by NZ (8.51 g/kg) and BC (8.24 g/kg). Total phosphorus (TP) content peaked in NZ (0.57 g/kg), followed by CH and BT. Total potassium (TK) content was highest in CH (9.17 g/kg), followed by NZ and YM, and lowest in BC (4.18 g/kg).

Regarding available nutrients, BT had the highest available phosphorus (AP) content among topsoil treatments (1.26 mg/kg, 250.0% increase over CK), followed by YM (1.02 mg/kg). ST subsoil had higher AP content (2.78 mg/kg) than the BT topsoil, consistent with vertical nutrient leaching under long-term cultivation. Available potassium (AK) content in BT reached 144.2 mg/kg, which is 65.3% greater than CK (87.25 mg/kg). YM (174.9 mg/kg) also maintained relatively high AK contents. ST subsoil had higher AK content (252.2 mg/kg) than BT topsoil. Soil alkali-hydrolyzable nitrogen (AN) content ranged from 0.044 to 0.483 g/kg in topsoil, with the highest value in YM being 11-fold that of the lowest in BC.

Overall, vegetation cover comprehensively improves soil nutrient status. Among all topsoil conditions, long-term *Z. mays* with straw return (BT) performs best in accumulating SOM and major available nutrients (AP, AK); two-year *Z. mays* (YM) showed advantages in improving AK and TN; and *L. lucidum* (NZ) excels in TP accumulation.

### 2.2. Changes in Soil Enzyme Activities Under Different Vegetation-Soil Conditions

**Figure 1:**
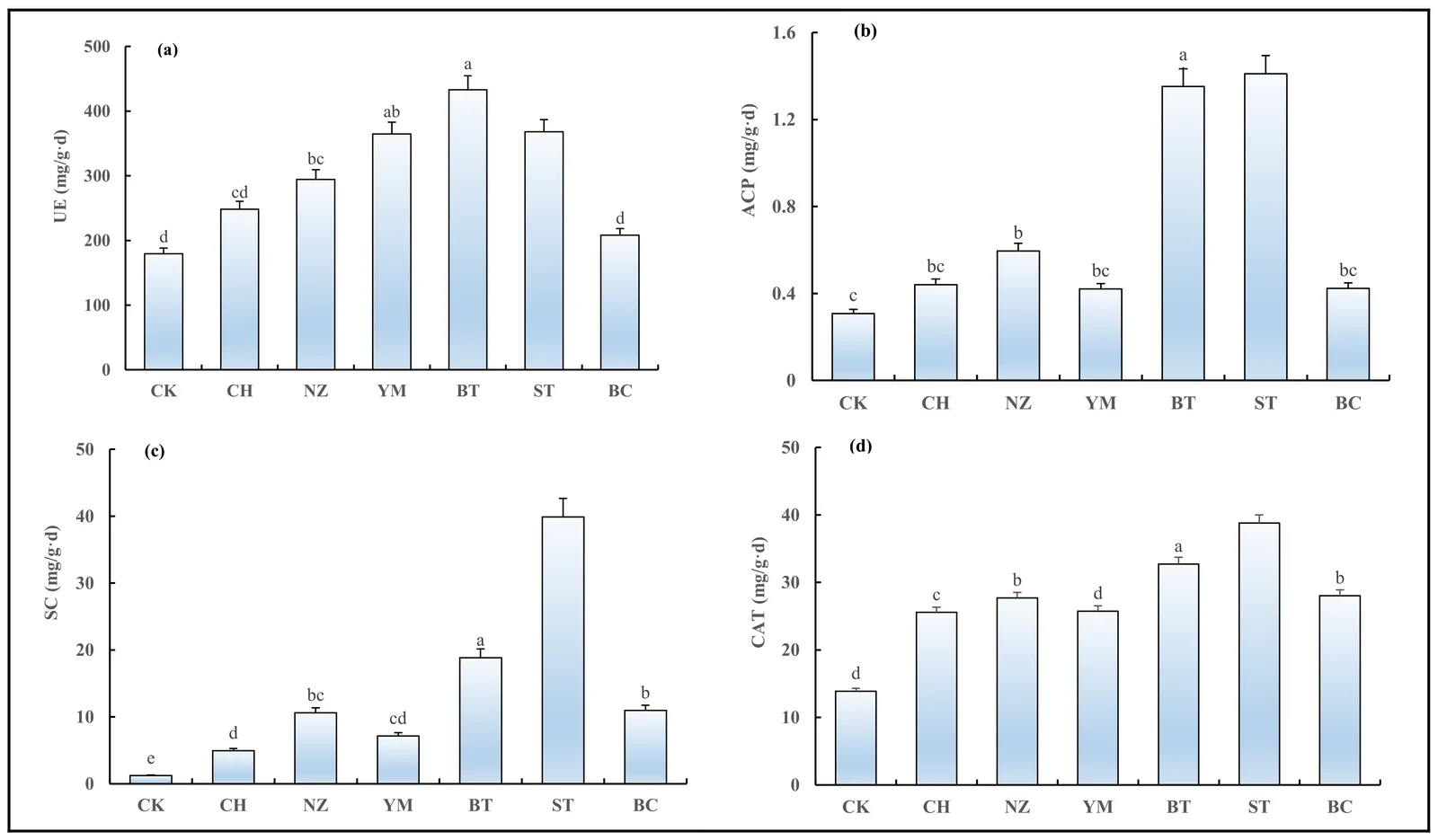
Soil enzyme activities under different vegetation-soil conditions in bauxite mine wasteland. Note: Different lowercase letters in the same column indicate significant differences among topsoil treatments (*p* < 0.05, Tukey’s HSD test); ST (10–20 cm subsoil) is shown for reference only and not included in the multiple comparison test; (**a**) urease; (**b**) alkaline phosphatase; (**c**) sucrase; (**d**) catalase.

As shown in Figure 1, vegetation cover conditions significantly altered the activities of urease (UE), alkaline phosphatase (ACP), sucrase (SC), and catalase (CAT). All cover conditions exhibited significantly higher enzyme activities than the bare control (CK) (*p* < 0.05), demonstrating that vegetation restoration was associated with promoted soil biochemical processes.

UE activity in topsoil ranked in the order BT > YM > NZ > CH > BC > CK (Figure 1a), with the BT pattern showing the highest activity (141.5% increase over CK). ACP activity decreased in the order BT > NZ > CH = BC = YM > CK (Figure 1b), with BT recording the highest activity among topsoil treatments. ST subsoil had higher phosphatase activity than BT topsoil, consistent with greater organic matter accumulation at depth. SC activity followed the order BT > BC > NZ > YM > CH > CK in topsoil (Figure 1c), with BT exhibiting the highest activity. ST subsoil showed extremely high sucrase activity, substantially greater than BT topsoil. CAT activity ranked in the order BT > BC > NZ > YM > CH > CK in topsoil (Figure 1d), with ST having higher activity than BT topsoil.

### 2.3. Effects of Vegetation-Soil Conditions on Soil Microbial Community Diversity

#### 2.3.1. Alpha Diversity of Soil Microbial Communities

Alpha diversity indices of soil bacterial communities are summarized in Table 2. After quality filtering and rarefaction to the same sequencing depth, 25,384 high-quality bacterial sequences and 41,206 high-quality fungal sequences were obtained per sample for subsequent analysis. Compared with CK, all five topsoil vegetation conditions showed higher bacterial alpha diversity, with statistically significant increases observed for the Shannon index in CH, NZ, YM, and BC (*p* < 0.05, Tukey’s HSD test). The ACE and Chao1 indices were significantly higher in NZ and YM compared with CK (*p* < 0.05). BT did not show significantly higher bacterial richness than CK after Tukey’s correction. No significant difference in the Simpson index was detected between most conditions and CK, but an overall upward trend in bacterial diversity was observed. Sequencing coverage exceeded 98% for all samples, confirming the reliability of the sequencing data.

For fungal communities, the Shannon index ranged from 3.52 to 4.89, with the YM condition showing the highest value, which was significantly higher than that of CK (*p* < 0.05). NZ, BT, and BC also had significantly higher Shannon indices than CK (*p* < 0.05). The Chao1 index followed the order NZ > YM > BC > ST > BT > CK > CH, with the NZ condition showing the highest value (871), a 108.9% increase over CK. The coverage index exceeded 98% across all samples, indicating sufficient sequencing depth.

#### 2.3.2. Beta Diversity of Soil Microbial Communities

**Figure 2:**
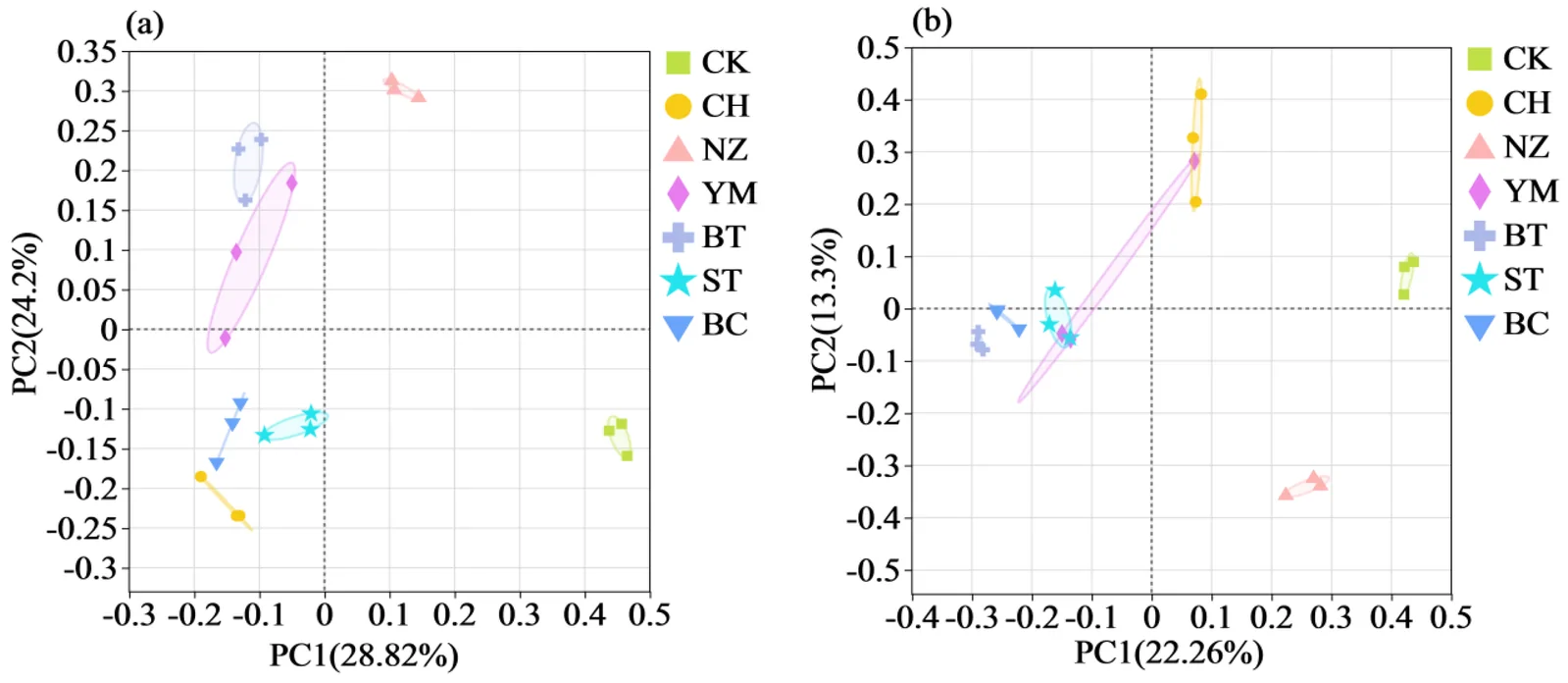
PCoA analysis of soil bacterial (**a**) and fungal (**b**) communities under different vegetation-soil conditions based on Bray–Curtis distance.

Principal coordinate analysis (PCoA) based on the Bray–Curtis distance revealed distinct clustering patterns for both bacterial (Figure 2a) and fungal communities (Figure 2b). For bacterial communities, the first two axes (PCoA1 and PCoA2) collectively explained 28.82% of the total variation. Soil bacterial communities from the vegetation conditions clustered mainly along the positive direction of PCoA1, with clear separation among conditions along the PCoA2 axis. The CK group concentrated along the negative direction of PCoA1, showing marked spatial separation from all revegetated patterns. PERMANOVA test confirmed that bacterial community structure differed significantly among conditions (R^2^ = 0.682, *p* = 0.001).

For fungal communities, PCoA1 explained 22.26% of the total community variation. Fungal communities in all revegetated conditions were clearly separated from the CK group. By contrast, fungal communities from the BT, BC, ST, YM, and CH patterns showed relatively tight clustering, while the NZ pattern formed a relatively independent cluster. PERMANOVA test confirmed significant differences in fungal community structure among patterns (R^2^ = 0.597, *p* = 0.001).

### 2.4. Composition of Soil Bacterial Communities Under Different Vegetation-Soil Conditions

In total, 682,459 raw bacterial 16S rRNA gene sequences were recovered from all samples, with an average read length of 415 bp. Clustering at 97% sequence similarity yielded 49,534 operational taxonomic units (OTUs), representing the major bacterial taxa in the test soils. The large number of OTUs is mainly due to the high heterogeneity of mine soil and the large number of low-abundance rare taxa under the 97% clustering threshold.

**Figure 3:**
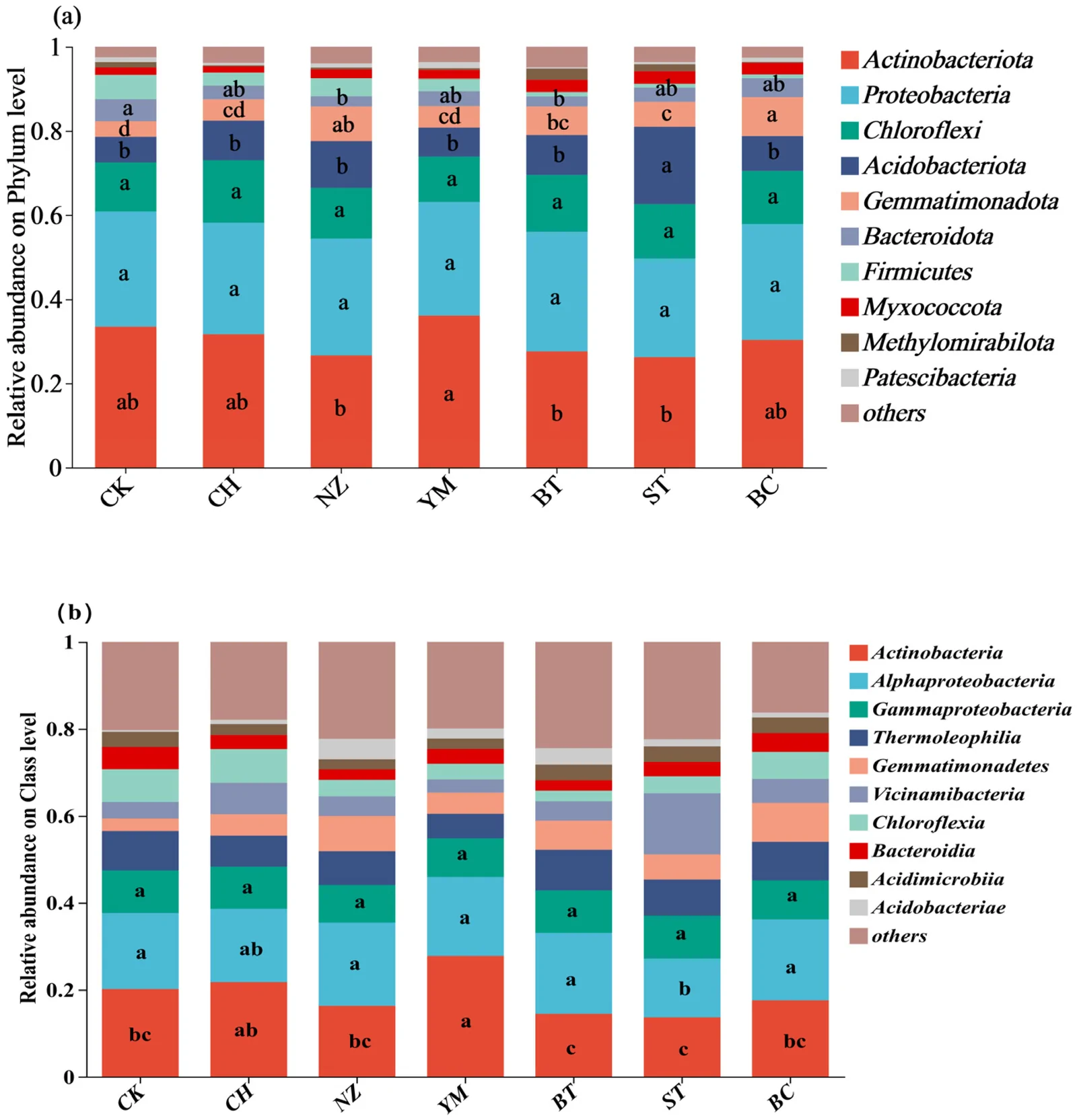
Relative abundance of dominant bacterial phyla (**a**) and bacterial classes (**b**) under different vegetation-soil conditions. Note: Different lowercase letters in the same column indicate significant differences (*p* < 0.05, Tukey’s HSD test).

At the phylum level (Figure 3a), six dominant phyla were identified in descending order of relative abundance: Actinobacteriota (28.15–35.22%), Chloroflexi (26.22–36.08%), Proteobacteria (23.40–28.39%), Acidobacteriota, Gemmatimonadota, and Bacteroidota, collectively accounting for over 75% of the total relative abundance. Their relative abundances in revegetated plots were significantly higher than in CK (*p* < 0.05), particularly in the BT and NZ patterns. Relative to CK, BT significantly increased the relative abundance of Acidobacteriota, while CH significantly enriched Actinobacteriota. Several bacterial phyla, including Chloroflexi and Proteobacteria, have been reported in previous studies to potentially harbor carbon-fixation-related genes (e.g., *cbbL*). Their relative enrichment in revegetated soils may suggest associations with soil carbon-cycling processes; however, this functional inference is based solely on taxonomic annotation and requires further verification through functional gene quantification and activity assays.

At the class level (Figure 3b), Alphaproteobacteria (13.58–19.21%) and Gammaproteobacteria (8.53–9.83%) were the dominant classes. All vegetation conditions significantly increased their relative abundances compared with CK (*p* < 0.05), with the most pronounced enrichment observed in ST and BT.

### 2.5. RDA of Dominant Bacterial Phyla and Soil Environmental Factors

**Figure 4:**
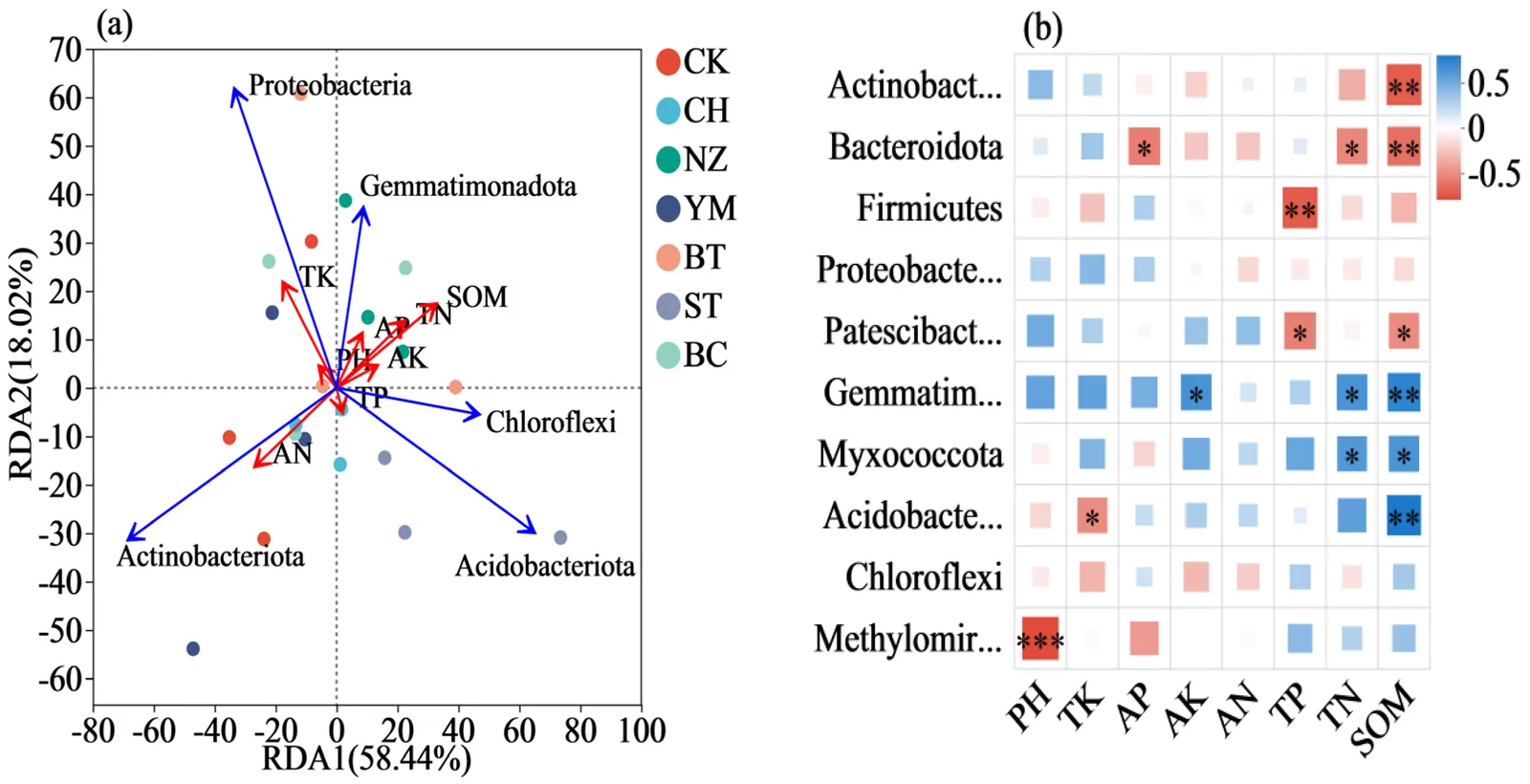
RDA ordination (**a**) and correlation heatmap (**b**) of dominant soil bacterial phyla and environmental variables. Monte Carlo permutation test: *p* = 0.002. Significance levels are based on FDR-adjusted *p*-values: * *p <* 0.05, ** *p* < 0.01, *** *p* < 0.001.

Before redundancy analysis (RDA), variance inflation factor (VIF) screening was performed to remove highly collinear variables (VIF > 10), and eight environmental factors (pH, SOM, TN, TP, TK, AK, AP, and AN) were finally retained for analysis. RDA revealed that the first two axes explained 76.46% of the total variation in bacterial community structure (axis 1: 58.44%; axis 2: 18.02%). The Monte Carlo permutation test confirmed that the selected environmental factors had a significant overall explanatory effect on bacterial community variation (*p* = 0.002). Axis 1 was negatively correlated with Actinobacteriota, Proteobacteria, pH, and TK, but positively correlated with Gemmatimonadota, Chloroflexi, Acidobacteriota, AP, AK, TN, SOM, and TP. Axis 2 was positively associated with Proteobacteria, Gemmatimonadota, TK, pH, AP, AK, TN, and SOM and negatively associated with Actinobacteriota, Acidobacteriota, Chloroflexi, TP, and AN (Figure 4a).

Correlation heatmap analysis (Figure 4b) further showed that SOM had an extremely significant negative correlation with Actinobacteriota (*p* < 0.01, FDR-adjusted). TK was significantly negatively correlated with Acidobacteriota (*p* < 0.05, FDR-adjusted) and extremely significantly positively correlated with SOM (*p* < 0.01, FDR-adjusted). Gemmatimonadota showed significant positive correlations with AK and TN (*p* < 0.05, FDR-adjusted) and an extremely significant positive correlation with SOM (*p* < 0.01, FDR-adjusted). Bacteroidota was significantly negatively correlated with AP and TN (*p* < 0.05, FDR-adjusted) and extremely significantly negatively correlated with SOM (*p* < 0.01, FDR-adjusted). Taken together, the RDA ordination and correlation heatmap indicate that SOM, TN, and nutrient availability variables were important environmental correlates of bacterial community variation in this observational dataset.

### 2.6. Composition of Soil Fungal Communities Under Different Vegetation-Soil Conditions

For fungi, a total of 1,086,372 high-quality ITS rRNA gene sequences were obtained from all samples, with an average length of 261 bp. Clustering at 97% similarity generated 12,506 OTUs, covering the major fungal groups in the soil.

**Figure 5:**
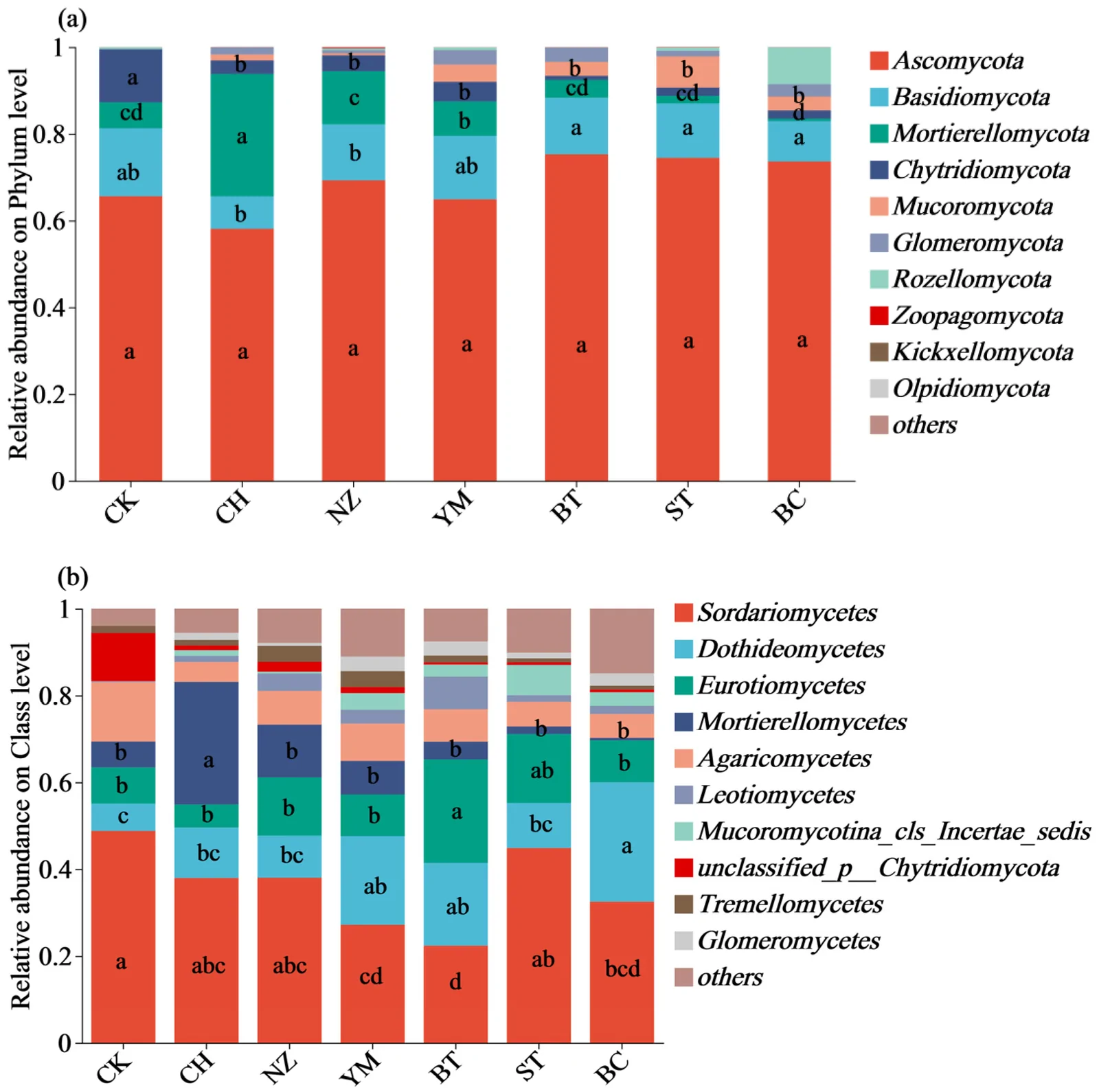
Relative abundance of dominant fungal phyla (**a**) and fungal classes (**b**) under different vegetation-soil conditions. Note: Different lowercase letters in the same column indicate significant differences (*p* < 0.05, Tukey’s HSD test).

At the phylum level (Figure 5a), four dominant phyla were identified in descending order: Ascomycota (58.08–75.28%), Basidiomycota (7.48–15.67%), Mortierellomycota (0.56–28.21%), and Chytridiomycota (0.94–12.25%). Compared with CK, the CH condition significantly increased the relative abundance of Mortierellomycota (*p* < 0.05). Ascomycota abundance was significantly elevated in the NZ, BT, ST, and BC conditions (*p* < 0.05). Basidiomycota abundance peaked in the YM and ST conditions, which were both significantly higher than in CK (*p* < 0.05).

At the class level (Figure 5b), Sordariomycetes (22.36–48.80%), Dothideomycetes (6.29–27.51%), Eurotiomycetes (5.32–23.31%), and Mortierellomycetes (0.56–28.21%) were the dominant classes. Relative to CK, the NZ, YM, and ST conditions significantly increased the relative abundance of Dothideomycetes (*p* < 0.05). The BT pattern harbored the highest Dothideomycetes abundance (27.51%), representing a 337.4% increase over CK. Dothideomycetes are commonly recognized as saprotrophic fungi involved in litter and organic matter decomposition [18]; their enrichment in BT may be related to the continuous input of straw carbon sources, although this functional interpretation remains speculative based on taxonomic inference alone and requires further verification.

### 2.7. RDA of Dominant Fungal Phyla and Soil Environmental Factors

**Figure 6:**
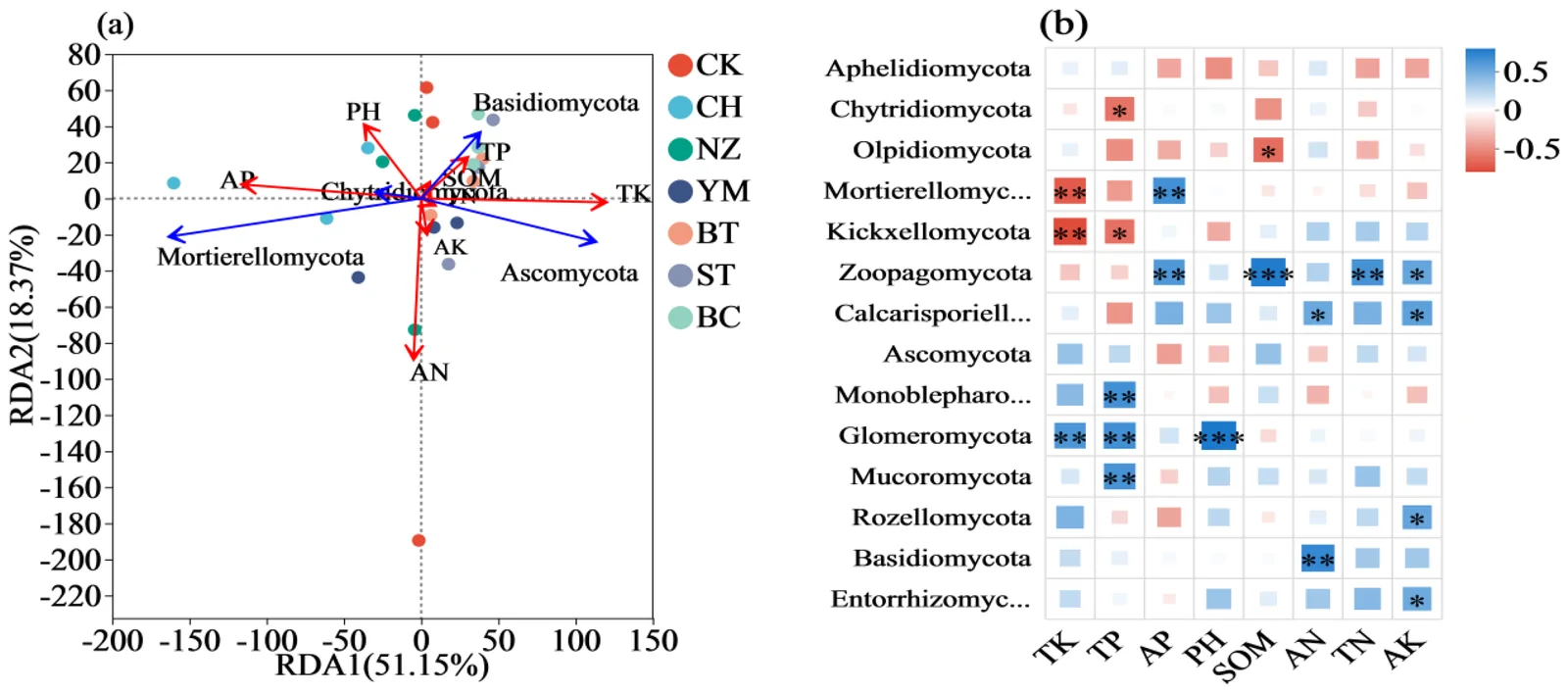
RDA ordination (**a**) and correlation heatmap (**b**) of dominant soil fungal phyla and environmental variables. Monte Carlo permutation test: *p* = 0.003. Significance levels are based on FDR-adjusted *p*-values: * *p* < 0.05, ** *p* < 0.01, *** *p* < 0.001.

After VIF screening, seven environmental factors were retained for fungal RDA. RDA results (Figure 6a) for fungal communities showed that the first two axes explained 69.52% of the total variation (axis 1: 51.15%; axis 2: 18.37%). The Monte Carlo permutation test confirmed that the selected environmental factors had a significant overall explanatory effect on fungal community variation (*p* = 0.003). Axis 1 was significantly positively correlated with AK, TK, TP, SOM, Ascomycota, and Basidiomycota (*p* < 0.05) and significantly negatively correlated with Mortierellomycota, Chytridiomycota, pH, AP, and AN (*p* < 0.05). Axis 2 was positively associated with TP, pH, AP, SOM, and Basidiomycota and significantly negatively associated with Mortierellomycota, Ascomycota, AK, TK, and AN (*p* < 0.05).

Correlation heatmap analysis (Figure 6b) revealed that AN had an extremely significant positive correlation with Basidiomycota (*p* < 0.01, FDR-adjusted). TK showed extremely significant negative correlations with Mortierellomycota and Kickxellomycota (*p* < 0.01, FDR-adjusted) and an extremely significant positive correlation with Glomeromycota (*p* < 0.01, FDR-adjusted). TP was significantly negatively correlated with Chytridiomycota and Kickxellomycota (*p* < 0.05, FDR-adjusted) and extremely significantly positively correlated with Glomeromycota and Mucoromycota (*p* < 0.01, FDR-adjusted). Overall, the fungal community pattern appeared to be correlated with multiple soil physicochemical factors, particularly nutrient-related variables, although these relationships should be interpreted cautiously within the observational scope of the present study.

## 3. Discussion

### 3.1. Soil Improvement Effects of Different Functional Vegetation Types and Potential Mechanisms

#### 3.1.1. Differential Responses of Soil Physicochemical Properties to Functional Vegetation Types

Vegetation cover serves as a critical pathway for ameliorating degraded mine soil, and its restoration effect is strongly modulated by plant functional traits and plant–soil feedback processes [19]. In this observational study, all vegetation conditions effectively neutralized the strongly acidic mine soil and elevated soil nutrient contents, yet the improvement effects differed significantly across functional types: long-term *Z. mays* cropland with straw return (BT) exhibited the strongest SOM and available nutrient accumulation among topsoil treatments, woody species (CH, NZ) performed superior performance in pH regulation and total nutrient improvement, and the pioneer herb *M. sinensis* (BC) presented a moderate overall improvement effect.

We hypothesize that the prominent nutrient accumulation in long-term *Z. mays* pattern stems from a positive feedback cycle of “fertilization input–high biomass production–straw return–organic matter accumulation”. Specifically, long-term no-till cultivation with annual straw return continuously inputs large amounts of organic carbon into the soil, which not only directly increases SOM content, but may also trigger the priming effect: fresh organic carbon stimulates microbial activity, accelerates the decomposition of native soil organic matter, and releases more available nutrients to further promote crop growth. This explains why available nutrients in BT increased far more than total nutrients. It should be noted that ST represents the 10–20 cm subsoil of the same long-term maize stand, and its higher nutrient level compared with the overlying BT topsoil likely reflects vertical leaching and accumulation processes under long-term straw return and fertilization. Since ST is sampled from a different soil depth, it is not statistically compared with topsoil treatments in this study; rather, the BT-ST paired data provide descriptive information on vertical nutrient distribution under long-term maize cultivation. The above mechanisms are inferred based on existing theories and have not been directly verified by process-level experiments in this study.

Targeting the unique habitat characteristics of karst bauxite wastelands—strong soil acidity, high active aluminum content, severe phosphorus fixation, and high erosion risk—the differential performance of vegetation types carries clear ecological significance and targeted restoration value. As a leguminous nitrogen-fixing tree, *R. pseudoacacia* significantly increased soil pH by 1.8–2.1 units compared with bare land, which effectively reduced exchangeable aluminum activity and may help alleviate aluminum toxicity to plant roots and soil microorganisms. This result aligns with the findings of Huang et al. (2025) in Guizhou bauxite reclamation areas, who reported that leguminous trees had the most significant acid-regulation effect on bauxite waste soil [2]. *L. lucidum* showed the strongest capacity to increase soil total phosphorus, which may be attributed to low-molecular-weight organic acids secreted by its root system [20]. These organic acids can chelate with aluminum and iron ions, release insoluble phosphate fixed by iron–aluminum oxides, and improve soil phosphorus availability. This finding supplements existing research on phosphorus activation by selected tree species in karst bauxite regions and provides a basis for selecting phosphorus-efficient restoration species. In comparison, the long-term maize straw return pattern mainly improves available nutrients by increasing organic matter input, with a weaker effect on total phosphorus accumulation than woody species, which is consistent with comparative studies of farmland reclamation and afforestation in Guangxi bauxite areas [21]. For the pioneer herb *M. sinensis*, although its improvement effect on individual soil indicators is moderate, its strong clonal reproduction and dense root network enable rapid soil surface coverage and reduced water erosion. This trait is particularly valuable for early-stage restoration of slope wastelands in karst mountainous areas with severe soil erosion. Collectively, these differential adaptation and improvement characteristics of selected species provide targeted options for karst bauxite restoration with varying degradation degrees and restoration objectives and supplement localized baseline data for regional mine rehabilitation practice [2,5].

#### 3.1.2. Coupling Response of Soil Enzyme Activities to Vegetation and Nutrients

Soil enzymes are the link between soil properties and microbial communities, and their activities directly reflect the intensity of soil nutrient transformation [22,23]. In this study, long-term *Z. mays* patterns had the highest enzyme activities, which is consistent with the trend of SOM and available nutrients. This result may be explained by two potential mechanisms: first, high SOM content provides abundant substrates for hydrolases such as sucrase and urease, directly promoting enzyme secretion; second, fertilization and root exudates stimulate microbial metabolic activity, and microorganisms release more extracellular enzymes to decompose organic matter and obtain nutrients [24].

From the perspective of soil biogeochemical coupling, the changes in enzyme activities form a complete chain with microbial communities and nutrient dynamics: vegetation restoration increases SOM and nutrient availability, which may promote the growth of copiotrophic microbial groups such as Proteobacteria; these microorganisms may secrete more sucrase, urease, and phosphatase, which may in turn accelerate organic matter decomposition and nutrient mineralization, potentially further improving soil fertility and forming a positive plant–soil feedback [25,26]. This coupling relationship may explain why the three indicators of physicochemical properties, enzyme activities, and microbial diversity show consistent improvement trends across conditions.

### 3.2. Vegetation Restoration Correlates with Soil Microbial Community Assembly: Environmental Filtering Hypothesis

#### 3.2.1. Vegetation Restoration Improves Microbial Diversity and Reshapes Community Structure

Soil microorganisms underpin vegetation restoration by mediating biogeochemical cycling and sustaining ecosystem functionality, which in turn promotes soil structural stability [27]. Our results showed that all vegetation restoration conditions increased soil bacterial and fungal alpha diversity and significantly shifted community structure, which is consistent with previous findings in mine restoration areas [28]. This may be because vegetation input of litter and root exudates may alleviate the nutrient limitation of mine soil, provide diversified carbon sources for microorganisms, and support the survival of more taxa [29].

For bacterial communities, Actinobacteriota, Chloroflexi, and Proteobacteria were the dominant phyla. Previous studies have reported that some taxa within Chloroflexi and Proteobacteria may carry carbon-fixation-related genes (e.g., *cbbL*) [30]. The relative enrichment of these phyla in revegetated soils suggests potential associations with soil carbon cycling, but this functional inference is based solely on taxonomic annotation and requires verification by subsequent functional gene quantification and activity assays. Actinobacteriota was the most abundant bacterial phylum, which is related to its strong stress tolerance and ability to decompose refractory organic matter, making it dominant in barren mine soils [31]. This is consistent with the results of microbial community surveys in karst bauxite wastelands reported by Hao et al. (2023), who found that Actinobacteriota was the absolute dominant flora in degraded bauxite soil due to its strong tolerance to aluminum toxicity and oligotrophic stress [32]. With the improvement of soil nutrients after vegetation restoration, the relative abundance of copiotrophic groups such as Proteobacteria increased significantly, and the microbial community structure gradually transformed from an oligotrophic type to a copiotrophic type, which reflected the improvement of soil ecological function from the perspective of microorganisms. The relative enrichment of Alphaproteobacteria in restoration treatments is consistent with the increase in TN, as this class contains many taxa involved in soil nitrogen cycling [33].

For fungal communities, Ascomycota was absolutely dominant, which is consistent with its wide ecological adaptability and role as the main decomposer of soil litter. Dothideomycetes, a class of predominantly saprotrophic fungi involved in litter decomposition [34], was relatively enriched in long-term *Z. mays* treatments, which may be related to the continuous input of straw carbon sources. This functional interpretation is based on taxonomic annotation and requires further experimental verification.

#### 3.2.2. Differential Driving Patterns of Bacterial and Fungal Communities: Multiple Possible Explanations

RDA results showed that bacterial communities were mainly correlated with SOM and TN, while fungal communities were more strongly correlated with AN. One possible explanation comes from the life history strategies of microorganisms: most soil bacteria are considered copiotrophs, which may grow rapidly when resources are abundant and are highly sensitive to total organic carbon and total nitrogen levels [35]; hence, SOM and TN may be the main correlates of bacterial community variation. In contrast, fungi have stronger hyphal foraging ability and can maintain growth under low-nitrogen conditions by decomposing refractory organic matter [36]; thus, they have a stronger competitive advantage in nitrogen-limited environments. When alkali-hydrolyzable nitrogen content changes, the competitive balance between fungi and bacteria shifts, potentially leading to significant changes in fungal community structure.

However, alternative explanations cannot be ruled out. For example, soil pH is a key driver of microbial community composition in acidic mine soils, and pH changes caused by different vegetation types may also lead to the differential responses of bacteria and fungi. In addition, soil moisture and substrate quality differences may also contribute to the observed pattern. The specific driving mechanism needs to be further verified by controlled experiments.

### 3.3. Application Implications and Limitations of Different Restoration Conditions

#### 3.3.1. Potential Application Scenarios for Different Restoration Conditions

The results of this observational study show that different restoration conditions have distinct advantages and potential applicable scenarios, and restoration schemes may be selected according to restoration objectives and site conditions. The following implications are preliminary observations based on this single-site study and need to be verified by multi-site long-term experiments before large-scale application.

For the goal of rapid soil improvement and agricultural land reclamation, long-term *Z. mays* cultivation with no-till straw return shows promising performance. It can rapidly increase soil organic matter and available nutrient contents in a short period and may improve soil physical structure through root penetration, achieving efficient improvement of mine soil. It should be clarified that this pattern relies on supporting fertilization management, and the improvement effect is the synergistic result of crop growth and exogenous nutrient input.

For the goal of long-term ecological restoration and ecosystem self-stability, woody species *R. pseudoacacia* and *L. lucidum* may be more suitable. Although their short-term improvement effect is not as fast as that of agricultural patterns, they do not require continuous artificial management after planting and may gradually improve soil fertility through nitrogen fixation and litter input, potentially forming a self-sustaining forest ecosystem.

For the initial stage of severely degraded wasteland restoration, *M. sinensis* is considered an ideal pioneer species. It can rapidly colonize barren and acidic soil, form continuous vegetation cover, reduce soil erosion, and create basic soil conditions for subsequent woody plant colonization.

#### 3.3.2. Limitations of the Study

Several limitations of this study should be acknowledged. First, this investigation was conducted at a single site and followed an observational design rather than a fully controlled experimental framework. The treatments varied in restoration age, management practices, and sampling depth, making it impossible to isolate the independent contributions of vegetation species, fertilization, straw return, or restoration duration. Furthermore, the absence of pre-restoration baseline soil data means that the observed differences in soil properties may partly reflect inherent spatial heterogeneity of the mine substrate rather than treatment effects alone. Second, most treatments represent only short-term (two-year) vegetation cover, and thus long-term restoration trajectories and ecosystem stability remain to be confirmed through continuous in situ monitoring. The ST treatment, which involved subsoil at 10–20 cm depth, introduces confounding between soil depth and planting duration; therefore, direct comparisons with topsoil treatments should be interpreted with caution. Third, the plant selection was limited to four selected species, which were all established as monocultures. Future work should expand the species pool and test mixed planting designs with optimized combinations to identify more effective restoration candidates. Fourth, in the long-term maize treatment, the independent effects of fertilization versus vegetation growth were not separated; controlled experiments are needed to quantify their respective roles. Fifth, carbon-cycling-related functions of microorganisms were inferred from taxonomic annotation alone; subsequent studies should incorporate functional gene quantification (e.g., *cbbL*) and activity assays to corroborate these inferences. Sixth, sampling was performed in a single season, so seasonal dynamics of soil enzyme activities and microbial communities were not captured; accordingly, our results are representative only of the conditions at the time of sampling. Seventh, the sample size was relatively modest, which warrants prudence in interpreting multivariate analyses such as redundancy analysis (RDA). Future studies with larger sample sizes and multi-site replicates would enhance the robustness and generalizability of the conclusions.

### 3.4. Restoration Implications and Outlook

Based on the observational findings of this study, we propose the following preliminary restoration considerations for karst bauxite mine wastelands: long-term *Z. mays* cultivation with straw return may be considered for rapid improvement of reclaimed mine farmland, woody species show potential for long-term ecological restoration, and M. sinensis could be explored for pioneer restoration in the initial stage. A phased mixed restoration strategy combining herbs and woody plants has potential application value.

It should be emphasized that the above considerations are based on single-site, single-season observational data, and there are confounding factors such as restoration age and management measures. The universality and causal relationships need to be further verified by multi-site, long-term controlled experiments. Future research should focus on disentangling the independent effects of vegetation, fertilization and straw return, verifying microbial carbon-cycling functions at the molecular level, and optimizing mixed-species restoration patterns.

## 4. Materials and Methods

### 4.1. Study Area

The study area is located in Daguzuo Village, Qingzhen City, Guizhou Province, China. It belongs to the subtropical humid monsoon climate zone with plateau mountain characteristics. The climate is mild and humid with abundant rainfall and distinct seasons. The mean annual temperature ranges from 13.5 to 14.5 °C, and annual precipitation is 1100–1300 mm, approximately 70% of which occurs from May to September. The average annual sunshine duration is 1100–1200 h, and the average frost-free period is 270 days. The main soil type is yellow earth (classified as Ferric Acrisol in the WRB soil classification system) with a pH of 6.5–7.0, suitable for the growth of diverse plant species.

The bauxite mine in this area has been in operation for over 60 years. Over the past decade, local authorities and mining enterprises have carried out a series of vegetation restoration practices in the abandoned mining area, including afforestation with selected tree species, grass planting for slope protection, and reclamation of cultivated land with straw return. The long-term *Z. mays* cropland has been cultivated for more than 15 years, adopting a no-till farming system with full straw return and conventional NPK fertilization management (annual rate: 225 kg N ha^−1^, 90 kg P_2_O_5_ ha^−1^, 120 kg K_2_O ha^−1^). In the last two years, *R. pseudoacacia*, *L. lucidum*, and *M. sinensis* were introduced for restoration trials with no artificial fertilization or irrigation after planting and under natural growth conditions. 

### 4.2. Sample Collection

Soil sampling was conducted in August 2024 across six topsoil (0–10 cm) vegetation-soil conditions: CK (unvegetated bare land), CH (two-year *R. pseudoacacia* plantation), NZ (two-year *L. lucidum* plantation), YM (two-year *Z. mays* cropland), BT (0–10 cm topsoil of >15-year *Z. mays* cropland), and BC (two-year *M. sinensis* grassland). Additionally, paired 10–20 cm subsoil samples (ST) were collected from the long-term *Z. mays* plots.

For each pattern, three 20 m × 20 m replicate plots were set up. Plots were arranged using a spatially independent random block design, with a minimum interval of 50 m between any two replicate plots to ensure spatial independence and avoid spatial autocorrelation. All plots were located in the same mining wasteland with relatively consistent parent material and terrain background. In each plot, five soil cores were collected via an S-shaped sampling strategy and mixed into one composite sample (~1000 g). Plant roots and gravel were removed on-site. Each composite sample was homogenized and divided into two subsamples: one portion was air-dried at room temperature, ground, and passed through a 100-mesh sieve for analysis of soil physicochemical properties; the other portion of fresh soil was sealed in sterile bags, stored in foam boxes with ice packs, transported to the laboratory within 12 h, and stored at 4 °C for enzyme activity determination and at −80 °C for high-throughput sequencing. DNA extraction was completed within 72 h of sampling.

### 4.3. Analytical Methods

#### 4.3.1. Soil Physicochemical Properties

Soil properties were determined following the methods described by Tang et al. [37]. Soil pH was measured with a pH meter at a soil: water ratio of 1:2.5. SOM was determined by the potassium dichromate–sulfuric acid colorimetric method. TN was measured by the Kjeldahl method. TP was determined by alkali fusion–molybdenum antimony anti-spectrophotometry, and TK by alkali fusion–flame photometry. AN was measured by the alkali hydrolysis diffusion method, AP by spectrophotometry, and AK by flame photometry.

#### 4.3.2. Soil Enzyme Activities

All enzyme activity assays were performed using fresh soil samples (stored at 4 °C) within one week of sampling. Results are expressed on a dry soil weight basis after moisture correction. Soil moisture content was determined gravimetrically by drying fresh soil at 105 °C for 24 h, and the conversion formula is: Dry soil weight = Fresh soil weight × (1 − moisture content). Four soil enzymes were assayed, with detailed units as follows:

Urease (UE): determined by phenol–hypochlorite colorimetry, unit: mg NH_3_-N g^−1^ 24 h^−1^; Alkaline phosphatase (ACP): determined by p-nitrophenyl phosphate disodium colorimetry, unit: mg p-nitrophenol g^−1^ 24 h^−1^; Sucrase (SC): determined by 3,5-dinitrosalicylic acid colorimetry, unit: mg glucose g^−1^ 24 h^−1^; Catalase (CAT): determined by ultraviolet spectrophotometry, unit: mg H_2_O_2_ g^−1^ h^−1^. All enzyme activity assays were performed according to the standard procedures described by Lu [38].

#### 4.3.3. Soil DNA Extraction and PCR Amplification

Total microbial DNA was extracted from 0.5 g fresh soil using the FastDNA^®^ Spin Kit for Soil (MP Biomedicals, Santa Ana, CA, USA). DNA integrity was checked by 1% agarose gel electrophoresis, and purity (A260/A280 ratio of 1.8–2.0) and concentration were determined using a NanoDrop 2000 spectrophotometer (Thermo Fisher Scientific, Waltham, MA, USA).

The V3–V4 region of the bacterial 16S rRNA gene was amplified using primers 338F and 806R. The ITS1–ITS2 region of the fungal rRNA gene was amplified using primers ITS1F and ITS2R. PCR was performed in a 25 μL reaction system with the following thermal program: initial denaturation at 95 °C for 3 min; 30 cycles of denaturation at 95 °C for 30 s, annealing at 55 °C for 30 s, and extension at 72 °C for 45 s; and a final extension at 72 °C for 10 min.

#### 4.3.4. High-Throughput Sequencing and Bioinformatics Analysis

PCR products were verified by agarose gel electrophoresis, and sequencing libraries were constructed using the NEXTFLEX Rapid DNA-Seq Kit. Sequencing was performed on the Illumina MiSeq PE300 platform.

Raw sequences were quality-controlled using Trimmomatic software version 0.39 (quality threshold Q20, minimum read length 200 bp), and chimeras were removed using the UCHIME algorithm. OTU clustering was performed at 97% sequence similarity. Bacterial OTUs were annotated against the Silva 138 database, and fungal OTUs against the UNITE 8.0 database. All samples were rarefied to the same sequencing depth (25,384 reads per sample for bacteria, 41,206 reads per sample for fungi) for alpha and beta diversity analysis to ensure comparability.

### 4.4. Data Processing

Data processing was performed using Excel 2019 and SPSS 24.0. Before statistical analysis, the Shapiro–Wilk test was used to test the normality of data, and Levene’s test was used to test the homogeneity of variances. One-way analysis of variance (ANOVA) followed by Tukey’s HSD test was used for multiple comparisons among treatments. Tukey’s HSD test was adopted to control the family-wise error rate for multiple comparisons. For indicators that did not satisfy normality or homogeneity of variance, the Kruskal–Wallis non-parametric test was used. Graphs were generated using Origin 2022.

Microbial community composition analysis and principal coordinate analysis (PCoA) based on Bray–Curtis distance were performed on the Majorbio cloud platform. Permutational multivariate analysis of variance (PERMANOVA) with 999 permutations was used to test the significance of community structure differences among treatments. Redundancy analysis (RDA) was performed after variance inflation factor (VIF) screening to remove highly collinear variables (VIF > 10) and combined with Monte Carlo permutation test (999 permutations) to identify key environmental factors correlated with microbial community variation. Pearson correlation analysis was used to examine the relationships among soil properties, enzyme activities and microbial diversity indices, with Benjamini–Hochberg false discovery rate (FDR) correction applied to all correlation *p*-values to control for multiple testing.

## 5. Conclusions

### Main Observational Findings

(1) Vegetation cover effectively mitigates soil degradation in karst bauxite mine wastelands, neutralizing strongly acidic soil and significantly increasing soil organic matter and nutrient contents. Different functional types show distinct advantages: long-term *Z. mays* with straw return performed best in accumulating organic matter and available nutrients among topsoil treatments, woody species excel in pH regulation and total nutrient improvement, and the pioneer herb *M. sinensis* shows strong stress tolerance.
(2) All vegetation cover treatments were associated with significantly enhanced soil urease, alkaline phosphatase, sucrase, and catalase activities, suggesting strengthened soil biochemical processes and nutrient transformation capacity. Enzyme activities are highly coupled with soil nutrient contents, jointly reflecting the degree of soil ecological function recovery.
(3) Vegetation cover significantly increases soil bacterial and fungal diversity and reshapes community structure, with significant beta diversity differentiation between restored and bare soils. Several bacterial phyla associated with carbon cycling in prior studies are relatively more abundant in revegetated soils, but these functional inferences require further verification. Soil organic matter, total nitrogen, and available potassium are the main environmental correlates of bacterial community variation, while alkali-hydrolyzable nitrogen is closely correlated with dominant fungal groups.

## Figures and Tables

**Table 1:** Basic physical and chemical properties of soil under different vegetation-soil conditions in bauxite mine wasteland. Note: Different lowercase letters in the same column indicate significant differences among topsoil treatments (*p* < 0.05, Tukey’s HSD test); ST is presented for descriptive purposes only and is not included in the multiple comparison.

Vegetation-Soil Condition	pH	SOM (g/kg)	AP (mg/kg)	AN (g/kg)	TP (g/kg)	TN (g/kg)	TK (g/kg)	AK (mg/kg)
CK	5.67 ± 0.09 d	3.32 ± 0.08 e	0.36 ± 0.08 e	0.104 ± 0.12 b	0.32 ± 0.02 d	2.02 ± 0.06 d	7.58 ± 0.07 d	87.25 ± 0.90 d
CH	7.89 ± 0.01 b	6.44 ± 0.43 c	0.55 ± 0.43 d	0.050 ± 0.004 b	0.45 ± 0.06 b	7.52 ± 0.05 c	9.17 ± 0.15 a	90.19 ± 0.34 c
NZ	6.92 ± 0.04 c	9.22 ± 0.04 b	0.69 ± 0.01 c	0.046 ± 0.005 b	0.57 ± 0.08 a	8.51 ± 0.16 b	8.83 ± 0.11 b	88.92 ± 0.04 cd
YM	8.07 ± 0.13 a	5.30 ± 0.09 d	1.02 ± 0.06 b	0.483 ± 0.066 a	0.35 ± 0.05 cd	12.60 ± 0.44 a	8.65 ± 0.11 c	174.90 ± 1.50 a
BT	6.77 ± 0.10 c	17.55 ± 0.50 a	1.26 ± 0.12 a	0.062 ± 0.001 b	0.44 ± 0.06 bc	7.37 ± 0.13 c	6.43 ± 0.09 e	144.20 ± 0.17 b
ST	7.49 ± 0.16	38.87 ± 0.72	2.78 ± 0.11	0.206 ± 0.020	0.18 ± 0.01	8.84 ± 0.19	7.10 ± 0.04	252.20 ± 0.85
BC	7.74 ± 0.07 b	6.41 ± 0.09 c	0.44 ± 0.02 de	0.044 ± 0.005 b	0.43 ± 0.02 bc	8.24 ± 0.34 bc	4.18 ± 0.02 f	82.15 ± 1.60 e

**Table 2:** Alpha diversity indices of soil microbial communities under different vegetation-soil conditions. Note: Different lowercase letters in the same column indicate significant differences among topsoil treatments (*p* < 0.05, Tukey’s HSD test); ST is presented for descriptive purposes only and is not included in the multiple comparison.

Microorganisms	Vegetation-Soil Condition	Sequences	Shannon Index	Simpson Index	ACE Index	Chao Index	Coverage
Bacteria	CK	25,384	5.63 ± 0.08 d	0.0053 ± 0.0008 b	2410 ± 134 d	2374 ± 125 d	98.88%
	CH	25,384	6.23 ± 0.15 b	0.0140 ± 0.0030 ab	2814 ± 368 cd	2767 ± 370 cd	98.88%
	NZ	25,384	6.43 ± 0.08 a	0.0065 ± 0.0009 b	3312 ± 178 a	3273 ± 188 a	98.88%
	YM	25,384	6.09 ± 0.40 bc	0.0156 ± 0.0013 a	3123 ± 142 ab	3068 ± 119 ab	98.81%
	BT	25,384	6.07 ± 0.05 bc	0.0077 ± 0.0009 b	2599 ± 308 d	2561 ± 293 d	98.94%
	ST	25,384	6.40 ± 0.06	0.0048 ± 0.0010	3352 ± 67	3270 ± 75	98.91%
	BC	25,384	6.08 ± 0.09 bc	0.0076 ± 0.0012 b	2815 ± 441 cd	2745 ± 388 cd	98.91%
Fungi	CK	41,206	3.52 ± 0.06 e	0.02 ± 0.01 b	374 ± 27 d	417 ± 99 d	98.81%
	CH	41,206	3.96 ± 0.03 de	0.06 ± 0.03 ab	669 ± 303 cd	374 ± 27 d	98.81%
	NZ	41,206	4.75 ± 0.01 ab	0.03 ± 0.01 b	864 ± 53 a	871 ± 53 a	98.88%
	YM	41,206	4.89 ± 0.02 a	0.02 ± 0.01 b	801 ± 110 a	802 ± 112 ab	98.81%
	BT	41,206	4.81 ± 0.01 a	0.11 ± 0.06 a	563 ± 116 bc	563 ± 117 bc	98.94%
	ST	41,206	4.08 ± 0.07	0.08 ± 0.07	626 ± 150	626 ± 151	98.91%
	BC	41,206	4.52 ± 0.01 bc	0.03 ± 0.01 b	663 ± 311 abc	663 ± 309 abc	98.91%

## Data Availability

The original raw FASTQ sequencing reads cannot be recovered because of the limited data-retention period of the sequencing service provider and thus could not be submitted to the NCBI-SRA database. Further inquiries can be directed to the corresponding authors.

## References

[1] Fidanchevski E., Šter K., Mrak M., Rajacic M., Koszo B.D., Ipavec A., Teran K., Žibret G., Jovanov V., Aluloska N.S. (2024). Characterization of Al-containing industrial residues in the ESEE region supporting circular economy and the EU green deal. Materials.

[2] Huang M., Fan L., Pan Z., Cheng J., Yang S., Yan X., Sun D., Huang L. (2025). Spatiotemporal differentiation characteristics and restoration dynamics of soil nutrients in a typical karst-type bauxite mining area, Guizhou Province, China. Environ. Earth Sci..

[3] Hao X., Leung K., Wang R., Sun W., Li Y. (2010). The geomicrobiology of bauxite deposits. Geosci. Front..

[4] Xue S., Zhu F., Kong X., Wu C., Huang L., Huang N., Hartley W. (2016). A review of the characterization and revegetation of bauxite residues (red mud). Environ. Sci. Pollut. Res..

[5] Charan K., Banerjee S., Mandal J., Bhattacharyya P. (2025). Soil stressors on ecophysiology of bauxite mine impacted soil: Heavy metal-acidity-organic matter nexus. J. Environ. Qual..

[6] Xiang Y., Gong J., Zhang L., Zhang M., Chen J., Liang H., Chen Y., Fu X., Su R., Luo Y. (2025). Research progress of mine ecological restoration technology. Resources.

[7] Zhao Y., Yuan X., Ran W., Zhao Z., Su D., Song Y. (2025). The ecological restoration strategies in terrestrial ecosystems were reviewed: A new trend based on soil microbiomics. Ecol. Evol..

[8] Li H., Chen W., Zhang C., Liang H. (2024). Coupling relationship between soil properties and plant diversity under different ecological restoration patterns in the abandoned coal mine area of southern China. Ecol. Evol..

[9] Xue Y., Liu W., Feng Q., Zhu M., Zhang J., Wang L., Chen Z., Li X. (2025). The role of vegetation restoration in shaping the structure and stability of soil bacterial community of alpine mining regions. Plant Soil.

[10] Wang B., Chen C., Xiao Y.M., Chen K.Y., Wang J., Zhao S., Zhou G.Y. (2024). Trophic relationships between protists and bacteria and fungi drive the biogeography of rhizosphere soil microbial community and impact plant physiological and ecological functions. Microbiol. Res..

[11] Liu J., Zhang S., Li E., Zhu Y., Cai H., Xia S., Kong C. (2022). Effects of cubic ecological restoration of mining wasteland and the preferred restoration scheme. Sci. Total Environ..

[12] Aili A., Zhang Y., Lin T., Xu H., Waheed A., Zhao W., Kuerban A., Liu K., Dou H. (2024). Optimizing vegetation restoration: A comprehensive index system for reclaiming abandoned mining areas in arid regions of China. Biology.

[13] Heckenroth A., Rabier J., Dutoit T., Torre F., Prudent P., Laffont-Schwob I. (2016). Selection of native plants with phytoremediation potential for highly contaminated mediterranean soil restoration: Tools for a non-destructive and integrative approach. J. Environ. Manag..

[14] Li S., Shakoor A., Wubet T., Zhang N., Liang Y., Ma K. (2018). Fine-scale variations of fungal community in a heterogeneous grassland in inner mongolia: Effects of the plant community and edaphic parameters. Soil Biol. Biochem..

[15] Zhou Z., Zhang G., Hua J., Xue J., Yu C. (2024). Tree species selection for optimizing soil carbon storage: Insights from litter decomposition and bacterial community analysis in coastal ecosystems. J. Environ. Manag..

[16] Jiang Y., Whalen J.K. (2025). Plasticity of maize (*Zea mays* L.) roots in water-deficient and nitrogen-limited soil. Crop Sci..

[17] Nie G., Yang Z., He J., Liu A., Chen J., Wang S., Zhang X. (2021). Genome-wide investigation of the NAC transcription factor family in *Miscanthus sinensis* and expression analysis under various abiotic stresses. Front. Plant Sci..

[18] Tang L., Zhan L., Han Y., Wang Z., Dong L., Zhang Z. (2023). Microbial community assembly and functional profiles along the soil-root continuum of salt-tolerant *Suaeda glauca* and *Suaeda salsa*. Front. Plant Sci..

[19] Raiesi F., Salek-Gilani S. (2020). Development of a soil quality index for characterizing effects of land-use changes on degradation and ecological restoration of rangeland soils in a semi-arid ecosystem. Land Degrad. Dev..

[20] Wang Y., Tu H., Zheng J., Li X., Wang G., Guo J. (2025). Ecological stoichiometric characteristics of plant–litter–soil among different forest stands in a limestone region of China. Plants.

[21] Jiang X., Lin M., Zhang X., Yu G., Jiang P., Liu J. (2024). Pioneer plants promote soil formation in a mixture of bauxite tailings and red mud. J. Environ. Manag..

[22] Ding C., Wu L., Li F., Hu W., Zhang Z. (2026). Synergistic regulation of soil enzymes and microbial communities by biochar-supported nano-hydroxyapatite under cadmium stress. Land Degrad. Dev..

[23] Daunoras J., Kačergius A., Gudiukaitė R. (2024). Role of soil microbiota enzymes in soil health and activity changes depending on climate change and the type of soil ecosystem. Biology.

[24] Baldrian P. (2014). Distribution of extracellular enzymes in soils: Spatial heterogeneity and determining factors at various scales. Soil Sci. Soc. Am. J..

[25] Wan X., Yu Z., Wang M., Zhang Y., Huang Z. (2022). Litter and root traits control soil microbial composition and enzyme activities in 28 common subtropical tree species. J. Ecol..

[26] Wang J., Yang X., Huang S., Wu L., Cai Z., Xu M. (2025). Long-term combined application of organic and inorganic fertilizers increases crop yield sustainability by improving soil fertility in maize-wheat cropping systems. J. Integr. Agric..

[27] Coban O., De Deyn G.B., van der Ploeg M. (2022). Soil microbiota as game-changers in restoration of degraded lands. Science.

[28] Chen C., Liu Q., Du W., Wu S., Li L., Li H., Pang X., Yin C. (2025). Vegetation restoration alleviates microbial nitrogen limitation in abandoned spoils at early-stage: Implications for vegetation configuration on the Qinghai-Tibetan plateau. J. Environ. Manag..

[29] Zhao M., Liu J., Hu X.K., Shi W., Guo X.L., Zhang X.Y., Fang K. (2025). Plant-derived inputs drive soil microbial and nutrient dynamics in subalpine *Picea asperata* plantations. Plant Soil.

[30] Cai S., Cheng J., Wang Q., Fan L. (2026). Bioavailable metals and soil general properties co-mediate microbial communities in uncovered and vegetated areas during different seasons in an abandoned Mo-Ni mining area. J. Environ. Manag..

[31] Stevenson A., Hallsworth J.E. (2014). Water and temperature relations of soil *Actinobacteria*. Environ. Microbiol. Rep..

[32] Hao C., Du P., Ren J., Hu L., Zhang Z. (2023). Halophyte Elymus dahuricus colonization regulates microbial community succession by mediating saline-alkaline and biogenic organic matter in bauxite residue. Sci. Total Environ..

[33] Tsoy O.V., Ravcheev D.A., Čuklina J., Gelfand M.S. (2016). Nitrogen fixation and molecular oxygen: Comparative genomic reconstruction of transcription regulation in *Alphaproteobacteria*. Front. Microbiol..

[34] Zhang X., Yin Y., Du L., Xi F., Wang J. (2025). Metagenomics reveals the effects of long-term greenhouse vegetable cultivation on soil microbial communities and carbon cycle functions. Plant Soil.

[35] Fierer N., Bradford M.A., Jackson R.B. (2007). Toward an ecological classification of soil bacteria. Ecology.

[36] Aleklett K., Ohlsson P., Bengtsson M., Hammer E.C. (2021). Fungal foraging behaviour and hyphal space exploration in micro-structured Soil Chips. ISME J..

[37] Tang J., Zhang L., Zhang J., Ren L., Zhou Y., Zheng Y., Luo L., Yang Y., Huang H., Chen A. (2020). Physicochemical features, metal availability and enzyme activity in heavy metal polluted soil remediated by biochar and compost. Sci. Total Environ..

[38] Lu R.S. (2000). Soil Agrochemical Analysis Methods.

